# Evaluating the Link Between Preoperative Comorbidities and the Risk of Venous Thromboembolism in Patients Undergoing Urological Surgery

**DOI:** 10.7759/cureus.78724

**Published:** 2025-02-08

**Authors:** Konstantinos Douroumis, Konstantinos Kotrotsios, Napoleon Moulavasilis, Evangelos Fragkiadis, Panagiota Stratigopoulou, Ioannis Adamakis, Ioannis Anastasiou, Dionysios Mitropoulos

**Affiliations:** 1 First Department of Urology, National and Kapodistrian University of Athens, Athens, GRC; 2 Department of Anesthesiology, Laiko General Hospital of Athens, Athens, GRC

**Keywords:** comorbidities, complications, preoperative assessment, urologic surgery, venous thromboembolic events

## Abstract

Introduction: Taking into consideration both the increasing number of elderly patients undergoing surgery and the fragility of this particular category of patients, a decisive step towards more effectively balancing venous thromboembolism (VTE) and bleeding risk is the development of a reliable predicting tool. The aim of this study is to appraise the relationship between comorbidity assessment tools and VTE risk assessment models (RAMs) in patients undergoing urological procedures.

Methods: Data were prospectively collected during a 20-month period (March 2021-October 2022) including 136 urologic inpatients with a mean age of 66.4 (± 14.4) years. Patients’ medical records were reviewed in order to evaluate the comorbidities using the Age-Adjusted Charlson Comorbidity Index (AA-CCI), Cumulative Illness Rating Scale (CIRS), American Society of Anesthesiologists (ASA) score and Index of Co-Existent Diseases (ICED). Venous thromboembolism risk was assessed through the European Association of Urology (EAU) RAM, American Urological Association (AUA) RAM, and Caprini score.

Results: Statistical analysis indicated the presence of a statistically significant relationship between all comorbidity scores and VTE RAMs, with only the CIRS score showing no significant deviations across the EAU RAM risk groups (p=0.111). The positive Kendall’s Tau-b coefficient values manifest a positive monotonic relationship between the two variables, meaning that higher comorbidity scores correspond to higher VTE risk categories.

Conclusion: Comorbidity scores and VTE RAMs demonstrate a high degree of concordance. This finding suggests that EAU and AUA RAMs, irrespective of their simplicity, can effectively incorporate underlying health conditions and constitute reliable alternatives to the more complex Caprini score.

## Introduction

A significant proportion of patients undergoing urological surgery are elderly, with studies indicating that 65% of these procedures are performed in individuals over the age of 65 years [1]. This percentage is expected to rise in the coming decades [2].

While some studies have noted that older urological inpatients experience higher rates of delirium, ICU admissions, and mortality [3,4], current evidence underscores that therapeutic decisions should prioritize a combination of life expectancy, comorbid conditions, and overall health rather than age alone [5].

Several models have been developed to evaluate a patient’s comorbidities quantitatively. The Age-Adjusted Charlson Comorbidity Index (AA-CCI), Cumulative Illness Rating Scale (CIRS), and Index of Co-Existent Diseases (ICED) are among the most frequently used tools [6-8]. Additionally, the American Society of Anesthesiologists (ASA) score is a widely utilized pre-anesthesia comorbidity assessment model, which has been shown to correlate with surgical outcomes and morbidity [9,10].

Venous thromboembolism (VTE) is a common and serious complication among urological surgery patients, contributing significantly to perioperative morbidity and mortality [11-13]. Balancing the risk of VTE with the potential for postoperative bleeding is critical when considering thromboprophylaxis. Currently, VTE risk assessment models (RAMs) are an integral part of perioperative planning in urology.

This study aims to evaluate the relationship between comorbidity assessment tools and VTE RAMs in patients undergoing urological procedures.

## Materials and methods

This prospective study analyzed data collected over a 20-month period (March 2021-October 2022). All urological inpatients scheduled for surgery were eligible to participate. Informed consent was obtained, and the study was approved by the scientific and ethics committee of the Laiko General Hospital of Athens, where the study took place.

Patient medical records were reviewed to assess comorbidities using the following tools. The AA-CCI was developed in 1987 and is a reliable, user-friendly, and frequently used method of streamlining the estimation risk of death from comorbid diseases [6]. CIRS offers a comprehensive assessment of 14 organ systems and is commonly used in the assessment of geriatric patients [7]. The ASA score assesses the patients’ pre-anesthesia comorbidities and when it is used along with other patient- and surgery-specific factors is useful as a perioperative risk assessment tool [9,10]. Finally, the Index of Co-Existent Diseases (ICED) is a risk assessment model that includes the severity of the patient's functional impairment along with his physical impairment caused by the comorbidities [8].

VTE risk was evaluated using the EAU VTE [5], AUA VTE [14], and Caprini scores [15]. The EAU VTE RAM was developed by the European Association of Urology and is based on a review of the most relevant and high-quality evidence regarding urology, general surgery, and gynecology [5]. This model is rather simple and is based only on the patient’s BMI and age, along with personal and family history of VTE. The American Urological Association endorsed the RAM developed by Geerts et al. in its Best Practice Statement [14,16]. This model calculates the VTE risk considering the severity of surgery along with the patient’s factors that are known to predispose to VTE. The Caprini score is a well-studied VTE RAM that has been validated for non-orthopedic surgical patients. It is calculated using 30 different factors in men and 33 factors in women, making it precise in calculating VTE risk [15].

Data collection, extraction, and organization were performed using Microsoft Office (Microsoft, Redmond, Washington, DC, USA). Statistical analyses included the χ²-test, Fisher’s exact test, ANOVA, Multinominal Logistic Regression analysis, and Kendall’s Tau-b correlation for trend analysis. Analyses were conducted using Jamovi software version 2.3.28 [17].

## Results

The cohort included 136 patients, aged 20-96 years (mean age 66.4 ± 14.4 years), with 80.9% being male. The BMIs ranged from 19.1 to 43.5 (mean 27.4 ± 4.1). Major pelvic surgeries were performed in 25 (18.4%) patients, upper urinary tract endoscopic surgeries in 29 (21.3%) patients, lower urinary tract endoscopic surgeries in 32.4% of patients, operations involving the penis, urethra, and scrotum in nine (6.6%) patients, laparoscopic procedures in five (3.7%) patients and open upper urinary tract surgeries in 24 (17.6%) patients.

Regarding the ASA index, a statistically significant association was observed between the distribution of patients based on the ASA index and the various risk groups outlined by the EAU, AUA, and Caprini criteria (p=0.01, p<0.001, p=0.002, respectively). When calculating Kendall’s Tau-b correlation coefficient, in order to more thoroughly investigate these relationships, all relationships exhibited a consistent positively directed trend (Kendall’s Tau-b coefficient=0.284, 0.302, and 0.288 respectively) (Table 1).

**Table 1:** ASA index correlation with VTE RAMs. * Fisher’s exact test was used instead of a chi-square test for the Caprini model. VTE RAM: venous thromboembolism risk assessment model; ASA: American Society of Anesthesiologists; EAU: European Association of Urology; AUA: American Urological Association

VTE RAM	Categories	Mean ASA score (SD)	Chi-square value	p-value
EAU score	Low	1.846 (0.583)	13.3	0.01
	Medium	2.220 (0.582)		
	High	2.250 (0.707)		
AUA score	Low	1.286 (0.488)	25	<0.001
	Moderate	1.932 (0.561)		
	High	2.136 (0.594)		
	Highest	2.417 (0.669)		
Caprini score	Low	1 (0)	-*	0.002
	Moderate	1.75 (0.5)		
	Higher	1.733 (0.594)		
	Highest	2.088 (0.591)		

In the case of the AA-CCI, a statistically significant correlation was established between the distribution of patients according to these indices and the various risk groups based on the criteria of the three RAMs, while AA-CCI was proven to have a moderately positive linear association with all the RAM defined risk groups (Kendall’s Tau-b coefficient=0.328, 0.306 and 0.502, respectively) (Table 2). Similar results were observed when we compared the three VTE RAMs with the ICED. (Table 3).

**Table 2:** AA-CCI correlation with VTE RAMs. VTE RAM: venous thromboembolism risk assessment model; AA-CCI: Age-Adjusted Charlson Comorbidity Index; ASA: American Society of Anesthesiologists; EAU: European Association of Urology; AUA: American Urological Association

VTE RAM	Categories	Mean AA-CCI (SD)	Chi-square value	p-value
EAU	Low	3.321 (1.869)	57.5	<0.001
	Medium	4.940 (1.707)		
	High	5.125 (3.357)		
AUA	Low	0.286 (0.756)	98.7	<0.001
	Moderate	3.890 (2.125)		
	High	4.477 (1.422)		
	Highest	5.333 (1.826)		
Caprini	Low	0	104	<0.001
	Moderate	0.750 (0.957)		
	Higher	1.867 (1.302)		
	Highest	4.566 (1.752)		

**Table 3:** ICED correlation with VTE RAMs. * Fisher’s exact test was used instead of a chi-square test for the Caprini model. VTE RAM: venous thromboembolism risk assessment model; ICED: Index of Co-Existent Diseases; EAU: European Association of Urology; AUA: American Urological Association

VTE RAM	Categories	Mean ICED score (SD)	Chi-square value	p-value
EAU	Low	0.885 (0.624)	18.4	0.005
	Medium	1.160 (0.650)		
	High	1.375 (0.744)		
AUA	Low	0.143 (0.378)	37.1	<0.001
	Moderate	0.959 (0.655)		
	High	1.114 (0.538)		
	Highest	1.500 (0.674)		
Caprini	Low	0	-*	<0.001
	Moderate	0.25 (0.5)		
	Higher	0.667 (0.488)		
	Highest	1.124 (0.629)		

Finally, the mean CIRS score displayed significant differences among the various risk groups according to the AUA and Caprini criteria, but not among the EAU risk groups (p<0.001, p<0.001, p=0.111, respectively). However, correlation analysis revealed the presence of a meaningful relationship between all these variables. This indicates that even if no statistically significant deviation from independence is produced between the EAU risk group and the CIRS score a monotonic relationship exists, although this conclusion should be interpreted with caution. As CIRS values range from 0 to 56 the mean CIRS, we opted for the comparison of the mean CIRS score between the various VTE risk groups (Table 4).

**Table 4:** CIRS correlation with VTE RAMs. VTE RAM: venous thromboembolism risk assessment model; CIRS: Cumulative Illness Rating Scale; EAU: European Association of Urology; AUA: American Urological Association

VTE RAM	Categories	Mean CIRS (SD)	F-value	p-value
EAU	Low	5.936 (3.598)	2.24	0.111
	Medium	7.200 (3.659)		
	High	7.500 (2.726)		
AUA	Low	1.143 (1.676)	10.3	<0.001
	Moderate	6.014 (3.277)		
	High	7.455 (3.420)		
	Highest	9 (3.516)		
Caprini	Low	0 (0)	9.6	<0.001
	Moderate	2.500 (1.915)		
	Higher	4.800 (2.513)		
	Highest	7.088 (3.468)		

## Discussion

The analysis yielded the noteworthy finding that the comorbidity assessment models and VTE risk assessment models demonstrated a high degree of concordance, with the exception of the EAU RAM and CIRS scores. These results concur with previously published literature investigating the relationship between comorbidity status and VTE occurrences [18]. More specifically, it has been observed that patients assigned to higher-risk groups based on either ASA or CCI scores are more likely to experience VTE complications postoperatively [19-21]. In fact, ASA has already been introduced as a component in a novel perioperative thromboembolism RAM, with encouraging results [22]. Furthermore, it is important to highlight that CCI can serve not only as a predictor for VTE but also as a predictor of its short-term mortality, given that higher CCI values have been associated with higher rates of short-term mortality after an episode of VTE [23].

The EAU and AUA VTE risk assessment models stand out for their simplicity and ease of use compared to the more complex Caprini score and various comorbidity assessment models. Specifically, the EAU RAM requires just four factors for calculation: age, BMI, family history of VTE in a first-degree relative, and a prior history of VTE [5]. In contrast, the Caprini score involves 30 factors for men and 33 for women, offering greater precision but requiring considerably more time and effort to compute, leading to a low rate of model utilization [15].

These findings carry potential implications. One possibility is that simpler VTE RAMs, such as the EAU and AUA scores, could reliably replace the more comprehensive Caprini score for thromboprophylaxis decisions, even in patients with significant comorbidities. This is supported by the observation that the EAU and AUA scores effectively account for underlying health conditions, even in patients with multiple comorbidities. In fact, in a previous publication, we found that EAU and AUA VTE RAMs may be even more consistent in providing recommendations for urologic patients, as the Caprini model's strict adherence may lead to excessive prophylaxis recommendations [24].

Another implication is that VTE RAMs might potentially replace comorbidity assessment models in urologic patients. This substitution could streamline patient evaluations, as both VTE RAMs and comorbidity scores are routinely calculated for individuals undergoing elective urological surgeries. Comorbidity assessment models play a critical role in treatment planning, as demonstrated in conditions such as prostate cancer, where management decisions are guided by health status, comorbidities, and life expectancy in accordance with EAU guidelines [5]. Simultaneously, VTE RAMs are essential for assessing perioperative risk, given the significant contribution of VTE to postoperative morbidity [11-13].

However, our study has limitations. Given that the completion of the informed consent form was a prerequisite to be included in the study, there is a chance that more health-conscious patients agreed to participate, introducing a certain amount of selection bias into the study. Moreover, both the relatively small sample size and the conduction of the study in a single hospital constitute two features that can potentially restrict the external validity of the results. Also, since the calculation of VTE risk scores was based on data deriving from medical records, missing data or inconsistencies could potentially influence the results. Lastly, it should be highlighted that no postoperative thromboembolic events or overall survival data were recorded.

## Conclusions

Comorbidity assessment models and VTE risk assessment models show a high degree of alignment. The EAU and AUA VTE scores are simpler and more user-friendly than the Caprini model, yet they effectively predict and incorporate underlying health conditions, even in patients with substantial comorbidities. Moreover, EAU and AUA VTE scores are simpler than the most commonly used comorbidity assessment models. These findings suggest that the EAU and AUA scores may serve as reliable alternatives to the Caprini score. Finally, VTE RAMs might potentially be able to replace comorbidity assessment models in urologic patients, both of which are routinely calculated before major urological surgeries.
